# *N*′-[4-(Dimethyl­amino)benzyl­idene]benzohydrazide

**DOI:** 10.1107/S1600536809036460

**Published:** 2009-09-16

**Authors:** Chuansheng Cui, Qingan Meng, Yong Wang

**Affiliations:** a College of Chemistry and Chemical Engineering, Liaocheng University, Shandong 252059, People’s Republic of China

## Abstract

In the title mol­ecule, C_16_H_17_N_3_O, the two aromatic rings form a dihedral angle of 4.51 (18)°. In the crystal structure, inter­molecular N—H⋯O hydrogen bonds link mol­ecules related by translation along the *a* axis into ribbons.

## Related literature

For the biological properties of Schiff base ligands, see Bedia *et al.* (2006 ▶). For related crystal structures, see: Fun *et al.* (2008 ▶); Alhadi *et al.* (2008 ▶); Nie (2008 ▶).
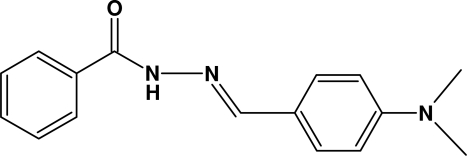


## Experimental

### 

#### Crystal data

C_16_H_17_N_3_O
*M* *_r_* = 267.33
Orthorhombic, 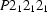

*a* = 5.131 (3) Å
*b* = 8.446 (4) Å
*c* = 32.502 (16) Å
*V* = 1408.5 (13) Å^3^
*Z* = 4
Mo *K*α radiation
μ = 0.08 mm^−1^
*T* = 298 K
0.40 × 0.31 × 0.15 mm

#### Data collection

Bruker SMART APEX CCD area-detector diffractometer
Absorption correction: multi-scan (*SADABS*; Sheldrick, 1996 ▶) *T*_min_ = 0.968, *T*_max_ = 0.988
6499 measured reflections
1489 independent reflections
768 reflections with *I* > 2σ(*I*)
*R*_int_ = 0.072

#### Refinement

*R*[*F* ^2^ > 2σ(*F* ^2^)] = 0.048
*wR*(*F* ^2^) = 0.066
*S* = 1.00
1489 reflections
183 parameters
H-atom parameters constrained
Δρ_max_ = 0.13 e Å^−3^
Δρ_min_ = −0.11 e Å^−3^

### 

Data collection: *SMART* (Siemens, 1996 ▶); cell refinement: *SAINT* (Siemens, 1996 ▶); data reduction: *SAINT*; program(s) used to solve structure: *SHELXS97* (Sheldrick, 2008 ▶); program(s) used to refine structure: *SHELXL97* (Sheldrick, 2008 ▶); molecular graphics: *SHELXTL* (Sheldrick, 2008 ▶); software used to prepare material for publication: *SHELXTL*.

## Supplementary Material

Crystal structure: contains datablocks I, global. DOI: 10.1107/S1600536809036460/cv2609sup1.cif

Structure factors: contains datablocks I. DOI: 10.1107/S1600536809036460/cv2609Isup2.hkl

Additional supplementary materials:  crystallographic information; 3D view; checkCIF report

## Figures and Tables

**Table 1:** Hydrogen-bond geometry (Å, °)

*D*—H⋯*A*	*D*—H	H⋯*A*	*D*⋯*A*	*D*—H⋯*A*
N1—H1⋯O1^i^	0.86	2.19	2.982 (4)	153

Symmetry code: (i) 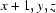.

**Fig. 1.**
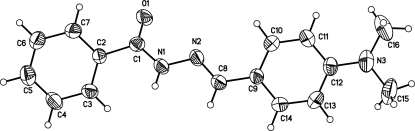
The molecular structure of (I) showing the atomic numbering scheme and 30% probability displacement ellipsoids.

## supplementary crystallographic information

### Comment

Schiff base ligands have received considerable attention during the last decades, mainly because of their structures or for their biological properties (Bedia *et al.*, 2006). We report here the crystal structure of the title new Schiff base compound, (I).

In (I) (Fig. 1), the bond lengths and angles are normal and comparable to the values observed in similar compounds (Nie *et al.*, 2008; Fun *et al.*, 2008; Alhadi *et al.*, 2008). The dihedral angle between the two aromatic rings in the Schiff base molecule is 4.51 (18) °, indicating that two these rings are approximately coplanar.

Weak intermolecular N—H···O hydrogen bonds (Table 1) link the molecules related by translation along axis *a* into ribbons.

### Experimental

Benzohydrazide (5.0 mmol), 20 ml ethanol and 4-(dimethylamino)benzaldehyde (5.0 mmol) were mixed in 50 ml flash. After refluxing 3 h, the resulting mixture was cooled to room temperature, and recrystalized from ethanol, and afforded the title compound as a crystalline solid. Elemental analysis: calculated for C_16_H_17_N_3_O: C 71.89, H 6.41, N 15.72%; found: C 71.63, H 6.55, N 15.64%.

### Refinement

All H atoms were placed in geometrically idealized positions (N—H 0.86 and C—H = 0.93–0.96 Å) and treated as riding on their parent atoms, with *U*_iso_(H) = 1.2–1.5 *U*_eq_(C, N). In the absence of any significant anomalous scatterers in the molecule, 1489 Friedel pairs were merged before the final refinement.

### Crystal data

**Table:**

C_16_H_17_N_3_O	*D*_x_ = 1.261 Mg m^−^^3^
*M**_r_* = 267.33	Mo *K*α radiation, λ = 0.71073 Å
Orthorhombic, *P*2_1_2_1_2_1_	Cell parameters from 731 reflections
*a* = 5.131 (3) Å	θ = 2.5–19.0°
*b* = 8.446 (4) Å	µ = 0.08 mm^−^^1^
*c* = 32.502 (16) Å	*T* = 298 K
*V* = 1408.5 (13) Å^3^	Block, red
*Z* = 4	0.40 × 0.31 × 0.15 mm
*F*(000) = 568	

### Data collection

**Table:**

Bruker SMART APEX CCD area-detector diffractometer	1489 independent reflections
Radiation source: fine-focus sealed tube	768 reflections with *I* > 2σ(*I*)
graphite	*R*_int_ = 0.072
φ and ω scans	θ_max_ = 25.0°, θ_min_ = 2.5°
Absorption correction: multi-scan (*SADABS*; Sheldrick, 1996)	*h* = −6→5
*T*_min_ = 0.968, *T*_max_ = 0.988	*k* = −10→5
6499 measured reflections	*l* = −38→37

### Refinement

**Table:**

Refinement on *F*^2^	Primary atom site location: structure-invariant direct methods
Least-squares matrix: full	Secondary atom site location: difference Fourier map
*R*[*F*^2^ > 2σ(*F*^2^)] = 0.048	Hydrogen site location: inferred from neighbouring sites
*wR*(*F*^2^) = 0.066	H-atom parameters constrained
*S* = 1.00	*w* = 1/[σ^2^(*F*_o_^2^)]
1489 reflections	(Δ/σ)_max_ = 0.045
183 parameters	Δρ_max_ = 0.13 e Å^−^^3^
0 restraints	Δρ_min_ = −0.11 e Å^−^^3^

### Special details

**Table:**

Geometry. All e.s.d.'s (except the e.s.d. in the dihedral angle between two l.s. planes) are estimated using the full covariance matrix. The cell e.s.d.'s are taken into account individually in the estimation of e.s.d.'s in distances, angles and torsion angles; correlations between e.s.d.'s in cell parameters are only used when they are defined by crystal symmetry. An approximate (isotropic) treatment of cell e.s.d.'s is used for estimating e.s.d.'s involving l.s. planes.
Refinement. Refinement of *F*^2^ against ALL reflections. The weighted *R*-factor *wR* and goodness of fit *S* are based on *F*^2^, conventional *R*-factors *R* are based on *F*, with *F* set to zero for negative *F*^2^. The threshold expression of *F*^2^ > σ(*F*^2^) is used only for calculating *R*-factors(gt) *etc*. and is not relevant to the choice of reflections for refinement. *R*-factors based on *F*^2^ are statistically about twice as large as those based on *F*, and *R*- factors based on ALL data will be even larger.

### Fractional atomic coordinates and isotropic or equivalent isotropic displacement parameters (Å^2^)

**Table:**

	*x*	*y*	*z*	*U*_iso_*/*U*_eq_	
N1	0.8782 (6)	0.6093 (3)	0.16374 (9)	0.0668 (9)	
H1	1.0323	0.6000	0.1738	0.080*	
N2	0.8356 (6)	0.5902 (4)	0.12192 (9)	0.0661 (9)	
N3	1.0131 (7)	0.4725 (4)	−0.07166 (10)	0.0921 (12)	
O1	0.4487 (5)	0.6576 (3)	0.17584 (7)	0.0753 (8)	
C1	0.6729 (8)	0.6429 (4)	0.18839 (11)	0.0584 (11)	
C2	0.7361 (7)	0.6580 (4)	0.23326 (10)	0.0527 (10)	
C3	0.9403 (7)	0.5772 (4)	0.25149 (12)	0.0708 (12)	
H3	1.0500	0.5149	0.2355	0.085*	
C4	0.9818 (8)	0.5887 (5)	0.29326 (12)	0.0841 (13)	
H4	1.1166	0.5321	0.3055	0.101*	
C5	0.8242 (9)	0.6838 (5)	0.31681 (12)	0.0825 (13)	
H5	0.8557	0.6944	0.3448	0.099*	
C6	0.6207 (9)	0.7630 (4)	0.29898 (12)	0.0778 (13)	
H6	0.5119	0.8259	0.3150	0.093*	
C7	0.5770 (8)	0.7494 (4)	0.25716 (12)	0.0696 (12)	
H7	0.4379	0.8029	0.2452	0.084*	
C8	1.0327 (7)	0.5383 (4)	0.10171 (11)	0.0650 (12)	
H8	1.1840	0.5108	0.1157	0.078*	
C9	1.0231 (7)	0.5218 (4)	0.05744 (11)	0.0597 (11)	
C10	0.8437 (8)	0.5959 (4)	0.03323 (11)	0.0690 (11)	
H10	0.7180	0.6588	0.0458	0.083*	
C11	0.8400 (9)	0.5818 (4)	−0.00856 (11)	0.0784 (12)	
H11	0.7136	0.6358	−0.0235	0.094*	
C12	1.0201 (9)	0.4892 (5)	−0.02920 (12)	0.0674 (11)	
C13	1.2002 (8)	0.4120 (5)	−0.00492 (13)	0.0843 (13)	
H13	1.3229	0.3462	−0.0172	0.101*	
C14	1.2020 (7)	0.4303 (5)	0.03710 (12)	0.0813 (14)	
H14	1.3297	0.3786	0.0523	0.098*	
C15	1.2059 (9)	0.3741 (5)	−0.09163 (11)	0.1262 (19)	
H15A	1.2164	0.2740	−0.0777	0.189*	
H15B	1.3725	0.4257	−0.0906	0.189*	
H15C	1.1568	0.3574	−0.1198	0.189*	
C16	0.8855 (10)	0.5890 (4)	−0.09662 (11)	0.1149 (18)	
H16A	0.7058	0.5968	−0.0887	0.172*	
H16B	0.8963	0.5584	−0.1250	0.172*	
H16C	0.9690	0.6897	−0.0929	0.172*	

### Atomic displacement parameters (Å^2^)

**Table:**

	*U*^11^	*U*^22^	*U*^33^	*U*^12^	*U*^13^	*U*^23^
N1	0.052 (2)	0.097 (2)	0.052 (2)	0.0054 (19)	−0.0077 (19)	0.0059 (18)
N2	0.062 (2)	0.089 (2)	0.047 (2)	−0.001 (2)	−0.0067 (19)	−0.0006 (18)
N3	0.124 (3)	0.094 (3)	0.058 (2)	−0.008 (3)	0.015 (3)	−0.004 (2)
O1	0.0489 (16)	0.110 (2)	0.0666 (17)	0.0048 (17)	−0.0071 (16)	0.0070 (15)
C1	0.052 (3)	0.063 (3)	0.060 (3)	−0.001 (3)	0.000 (3)	0.004 (2)
C2	0.051 (3)	0.060 (3)	0.047 (2)	−0.004 (2)	0.001 (2)	0.001 (2)
C3	0.053 (3)	0.097 (3)	0.063 (3)	0.013 (2)	0.001 (2)	0.012 (2)
C4	0.074 (3)	0.117 (4)	0.062 (3)	0.008 (3)	−0.015 (3)	0.006 (3)
C5	0.086 (3)	0.104 (4)	0.058 (3)	−0.010 (3)	0.002 (3)	0.000 (3)
C6	0.086 (4)	0.082 (3)	0.066 (3)	0.012 (3)	0.007 (3)	−0.007 (2)
C7	0.072 (3)	0.068 (3)	0.069 (3)	0.015 (3)	0.003 (3)	0.005 (2)
C8	0.055 (3)	0.083 (3)	0.057 (3)	0.010 (3)	−0.003 (2)	0.005 (2)
C9	0.053 (3)	0.079 (3)	0.047 (2)	0.003 (3)	0.005 (2)	0.005 (2)
C10	0.070 (3)	0.075 (3)	0.062 (3)	0.015 (3)	0.004 (3)	−0.004 (2)
C11	0.091 (3)	0.089 (3)	0.056 (3)	0.009 (3)	−0.006 (3)	0.007 (2)
C12	0.078 (3)	0.069 (3)	0.056 (3)	−0.006 (3)	0.012 (3)	0.005 (2)
C13	0.081 (3)	0.097 (3)	0.075 (3)	0.021 (3)	0.020 (3)	−0.002 (3)
C14	0.067 (3)	0.111 (4)	0.066 (3)	0.032 (3)	0.002 (3)	0.006 (3)
C15	0.112 (4)	0.196 (5)	0.071 (3)	−0.019 (4)	0.028 (3)	−0.033 (3)
C16	0.180 (5)	0.099 (4)	0.066 (3)	−0.018 (4)	−0.021 (3)	0.014 (3)

### Geometric parameters (Å, °)

**Table:**

N1—C1	1.353 (4)	C7—H7	0.9300
N1—N2	1.386 (3)	C8—C9	1.446 (4)
N1—H1	0.8600	C8—H8	0.9300
N2—C8	1.283 (4)	C9—C10	1.363 (4)
N3—C12	1.388 (4)	C9—C14	1.370 (4)
N3—C16	1.433 (4)	C10—C11	1.363 (4)
N3—C15	1.446 (4)	C10—H10	0.9300
O1—C1	1.227 (4)	C11—C12	1.384 (5)
C1—C2	1.499 (4)	C11—H11	0.9300
C2—C7	1.366 (4)	C12—C13	1.379 (4)
C2—C3	1.384 (4)	C13—C14	1.374 (4)
C3—C4	1.378 (4)	C13—H13	0.9300
C3—H3	0.9300	C14—H14	0.9300
C4—C5	1.373 (4)	C15—H15A	0.9600
C4—H4	0.9300	C15—H15B	0.9600
C5—C6	1.369 (5)	C15—H15C	0.9600
C5—H5	0.9300	C16—H16A	0.9600
C6—C7	1.382 (4)	C16—H16B	0.9600
C6—H6	0.9300	C16—H16C	0.9600
			
C1—N1—N2	118.8 (3)	C10—C9—C14	115.6 (4)
C1—N1—H1	120.6	C10—C9—C8	123.6 (4)
N2—N1—H1	120.6	C14—C9—C8	120.8 (4)
C8—N2—N1	114.7 (3)	C11—C10—C9	123.0 (4)
C12—N3—C16	120.3 (4)	C11—C10—H10	118.5
C12—N3—C15	119.2 (4)	C9—C10—H10	118.5
C16—N3—C15	116.9 (4)	C10—C11—C12	121.5 (4)
O1—C1—N1	123.7 (3)	C10—C11—H11	119.2
O1—C1—C2	121.1 (4)	C12—C11—H11	119.2
N1—C1—C2	115.2 (3)	C11—C12—C13	115.9 (4)
C7—C2—C3	119.2 (3)	C11—C12—N3	121.5 (4)
C7—C2—C1	118.2 (4)	C13—C12—N3	122.5 (4)
C3—C2—C1	122.6 (4)	C14—C13—C12	121.3 (4)
C4—C3—C2	120.3 (4)	C14—C13—H13	119.4
C4—C3—H3	119.9	C12—C13—H13	119.4
C2—C3—H3	119.9	C9—C14—C13	122.6 (4)
C5—C4—C3	120.0 (4)	C9—C14—H14	118.7
C5—C4—H4	120.0	C13—C14—H14	118.7
C3—C4—H4	120.0	N3—C15—H15A	109.5
C6—C5—C4	120.0 (4)	N3—C15—H15B	109.5
C6—C5—H5	120.0	H15A—C15—H15B	109.5
C4—C5—H5	120.0	N3—C15—H15C	109.5
C5—C6—C7	120.0 (4)	H15A—C15—H15C	109.5
C5—C6—H6	120.0	H15B—C15—H15C	109.5
C7—C6—H6	120.0	N3—C16—H16A	109.5
C2—C7—C6	120.6 (4)	N3—C16—H16B	109.5
C2—C7—H7	119.7	H16A—C16—H16B	109.5
C6—C7—H7	119.7	N3—C16—H16C	109.5
N2—C8—C9	121.0 (4)	H16A—C16—H16C	109.5
N2—C8—H8	119.5	H16B—C16—H16C	109.5
C9—C8—H8	119.5		

### Hydrogen-bond geometry (Å, °)

**Table:**

*D*—H···*A*	*D*—H	H···*A*	*D*···*A*	*D*—H···*A*
N1—H1···O1^i^	0.86	2.19	2.982 (4)	153

Symmetry codes: (i) *x*+1, *y*, *z*.
